# Variation of bacterial communities in water and sediments during the decomposition of *Microcystis* biomass

**DOI:** 10.1371/journal.pone.0176397

**Published:** 2017-04-24

**Authors:** Dayong Zhao, Xinyi Cao, Rui Huang, Jin Zeng, Qinglong L. Wu

**Affiliations:** 1 State Key Laboratory of Hydrology-Water Resources and Hydraulic Engineering, College of Hydrology and Water Resources, Hohai University, Nanjing, China; 2 State Key Laboratory of Lake Science and Environment, Nanjing Institute of Geography and Limnology, Chinese Academy of Sciences, Nanjing, China; Jinling Institute of Technology, CHINA

## Abstract

The bacterial community composition in water and sediment samples during the decomposition of *Microcystis* biomass were analyzed using the 454 pyrosequencing technique. We found dramatic shifts in the bacterial community composition of water and sediments after the addition of *Microcystis* biomass. Among all the detected phyla, only *Firmicutes* was found to be dominant in both water and sediment samples. The genus *Clostridium sensu stricto* was the absolutely dominant group in *Firmicutes* and showed drastic variations with incubation time during the decomposition process. Peak values in relative abundance of *Clostridium sensu stricto* appeared in the first few days for water and sediment samples. Environmental factors such as pH, dissolved oxygen (DO), and dissolved organic carbon (DOC) in water samples showed drastic variations during the decomposing process, which might be the prominent forces driving the variation of bacterial communities. The abundant genus, *Clostridium sensu stricto*, were thought to be well adapted to higher DOC and turbidity and lower pH and DO conditions. Compared with the sediment samples, the decomposition of *Microcystis* biomass had greater influence on the bacterial community composition in water and *Clostridium sensu stricto* might play important roles in the process of *Microcystis* biomass decomposition.

## Introduction

Bacteria constitute a crucial component of aquatic ecosystems and play important roles in transforming phytoplankton-derived organic matter [1–2]. Cyanobacterial blooms, especially *Microcystis* blooms, are frequently found in eutrophic freshwater ecosystems and can pose notable risks to ecological and human health [3–5]. Bacterial communities associated with marine phytoplankton blooms [2, 6–7] and bloom-forming freshwater phytoplankton in growing stage [1, 8] have also received considerable attention. In contrast, only a few studies have examined the bacterial community composition in response to the breakdown of algal blooms in freshwater lakes [9–11]. Understanding the variations of bacterial community composition and diversity during the bloom decomposition process has critical implications for a better understanding of the resistance and resilience of bacterial communities in aquatic ecosystems.

The development and subsequent decomposition of *Microcystis* blooms always caused drastic environmental changes and seriously degraded water quality of the surrounding water [4, 12–13]. Elevated nutrient concentrations, reduced pH and dissolved oxygen (DO) concentrations were observed in *Microcystis* dominated water during the decomposition process [3, 14–15]. Variation of bacterial communities was significantly related to environmental factors, thus the physicochemical changes induced by decomposition of *Microsystis* blooms may have impacts on the bacterial communities in water column and sediments, and the changes in bacterial community composition could influence the decomposition process [16–17]. Several studies have examined the bacterioplankton or particle-attached bacterial communities in water but neglected the bacterial communities in the sediments during the decomposition of *Microcysti*s biomass [9–11]. Shao et al. [18] examined the sediment bacterial communities but the relationships between bacterial community and environmental factors were not well discussed. Furthermore, the bacterial communities in the previous studies were carried out by means of terminal restriction fragment length polymorphism (T-RFLP) or denaturing gradient gel electrophoresis (DGGE) followed by cloning and sequencing of selected samples. The resolution of these methods might ignore the rare species and result in the underestimation of species richness.

In the present study, laboratory incubation experiments were conducted. 454 pyrosequencing, was used to analyze the bacterial communities in water and sediment. The objectives of this study were (1) to examine the variation of bacterial community composition in water and sediments during the *Microcystis* decomposition process, and (2) to understand the potential effects of environmental factors on the bacterial communities.

## Materials and methods

### Experimental design

Lake Taihu (N 31°29′14″, E 120°12′41″) is a large shallow eutrophic freshwater lake located in eastern China and dominated by intensive cyanobacteria blooms, especially *Microcystis* blooms, which could form heavy scum in some areas of the lake [12, 19]. In the present study, the microcosms were constructed using lake water, sediments, and *Microcystis* assemblages collected from Lake Taihu (This study has been approved by the Taihu Basin Authority of the Chinese Government. The field sampling did not involve endangered, protected species and vertebrate animals.). The sediments used were fully mixed and sieved to remove large particles or macrobenthos. The *Microcystis* biomass were rinsed three times with distilled water and transported to laboratory quickly before use. The *Microcystis* spp. constituted up to 97% of the total phytoplankton cells by using microscopic examination. The collected *Microcystis* assemblages were frozen at -80°C and then freeze dried before adding to the microcosms.

The microcosms were constructed in plexiglass columns (15 cm in diameter and 35 cm long). The height of the sediments was 12 cm and the depth of the overlying water was 20 cm in the constructed microcosms. According to the observed *Microcystis* biomass in Lake Taihu in summer, three treatments were designed: High *Microcystis* biomass treatment (0.33 g/L), moderate *Microcystis* biomass treatment (0.17 g/L), and control treatment without addition of *Microcystis*. Each treatment had three replicates. The fresh Cyanobacteria were dried with a vacuum freeze dryer (ALPHA 1–2, CHRIST, Germany) and then added into the microcosms. The fresh cyanobacteria cells can survive or grow under dark anaerobic conditions [20], and the aim of this study was to examine the degradation of *Microcystis* biomass on bacterial communities, thus dried *Microcystis* biomass was added into the experimental systems. Deionized water was added to keep the water level during the experimental process.

The microcosms were incubated under darkness at 22.5±1°C. Water and sediment samples were collected from microcoms at 0, 2, 5, 10, and 20 d during the incubation period. Bacterioplankton in water samples were filtered with 0.22 μm membrane and stored at -80°C. Surface sediment samples (0–1 cm) were collected by the self-made sampler and stored in -80°C freezer until DNA extraction. The water and sediment samples were collected from three replicates and mixed into a single sample for bacterial community analysis.

### Physicochemical parameters measurement

Water chemistry parameters, including pH, oxidation reduction potential (ORP), dissolved oxygen (DO), and turbidity were measured at 0, 2, 5, 10, and 20 d with electrodes during the experimental process. Water samples were filtered through 0.45 μm membrane (Millipore, Billerica, MA, USA) and analyzed with TOC 5000A (Shimadzu, Japan) for dissolved organic carbon (DOC). Unfiltered water samples were used to measure total nitrogen (TN) and total phosphorus (TP) with spectrophotometrical methods [21]. Sediment samples were freeze dried and used for total nitrogen (TN), total phosphorus (TP), organic matter (OM), and pH measurement according to Jin and Tu [21].

### DNA extraction, PCR, and pyrosequencing

Based on the measured physicochemical data, the environmental conditions in *Microcystis* addition treatments showed dramatic changes on day 2 and day 5 and recovered on day 20. Thus 9 composited water samples and 7 mixed sediment samples on day 0 (represented pre-decomposition), day 2 and 5 (represented dramatic decomposition period), and day 20 (represented post-decomposition) were selected for bacterial community analysis. Another water sample at 40 d was collected from the incubation system to ensure the recovery of bacterial communities.

For water samples, genomic DNA was extracted using a standard phenol-chloroform extraction method [22]. For sediment samples, DNA was extracted using the PowerSoil DNA isolation kit (MoBio Laboratories, CA). The DNA extracted from the sediment samples were purified by the PowerSoil DNA Purification kit (MoBio Laboratories, CA) according to the manufacturer's instructions, quantified with a Nano-Drop ND-1000 spectrophotometer (NanoDrop Technologies, Wilmington, DE, USA), and then used as template for PCR.

The bacterial 16S rRNA genes were amplified with the universal primers 8F (AGAGTTTGATCCTGGCTCAG) and 533R (TTACCGCGGCTGCTGGCAC) with the Roche 454 sequencing adapters and a unique 10 bp barcode sequence [23–24]. The PCR mixture contained 0.4 μl FastPfu Polymerase (2.5 unit/μl), 4 μl 5×FastPfu buffer, 2 μl dNTPs (2.5 mM), 0.4 μl forward and reverse primer (5 μM), and 1 μl Gel-purified genomic DNA (about 10 ng/μl) in a volume of 20 μl. The PCR protocol was carried out as follows: an initial denaturation at 95°C for 2 min, 25 cycles of denaturation at 95°C for 30 s, annealing at 55°C for 30 s, and extension at 72°C for 1 min, with a final extension at 72°C for 10 min. To minimize the difference resulting from PCR amplification, the PCR reactions were carried out in triplicates. The PCR products for each sample were pooled and purified by gel electrophoresis.

The purified PCR products were quantified and more than 200 ng products were used for pyrosequencing. Pyrosequencing was carried out using a 454 FLX Titanium platform (Roche) at the Shanghai Majorbio Bio-Pharm Technology Co., Ltd. The obtained raw data have been deposited in the NCBI short-reads archive database (Accession Number: SRP061263) for all the water and sediment samples.

### Data processing and statistical analysis

The sequence reads were processed using Mothur (v 1.33.0) software package following the 454 standard operating procedure (SOP) (https://www.mothur.org/wiki/454_SOP). Low-quality sequences (average quality < 27) were discarded. Short sequences (< 200 bp after excluding the primer and barcode), containing ambiguous reads, or homopolymers > 8 nt were excluded from further analysis. The remaining sequences were aligned according to the SILVA 16S rRNA gene template using the nearest alignment space termination (NAST) algorithm. The command 'chimera.uchime' in Mothur was applied to remove putative chimeric sequences, and the command 'pre.cluster' was used to remove sequences that are likely due to pyrosequencing errors. The Bayesian classifier was used to classify the sequences against the Ribosomal Database Project 16S rRNA gene training set (http://rdp.cme.msu.edu).

The reads were clustered into operational taxonomic units (OTUs) at 3% dissimilarity and a pairwise distance matrix was calculated using Mothur. For diversity estimates and comparisons between samples, the sequences were randomly extracting equal numbers of sequences from each sample dataset to equally compare all samples at the same sequencing depth. Based on the OTU table and phylogenetic tree, bacterial diversity was estimated by the indices such as Shannon index (*H*) and the Faith’s phylogenetic diversity (Faith’s PD) [25–26]. The taxonomic diversity was calculated with the command 'summary.single' in Mothur. The Faith’s PD [25] was estimated with the 'picante' package in R 3.3.1 (https://www.r-project.org/).

To determine the dissimilarity of the bacterial communities between any pair of samples, the Bray-Curtis dissimilarity matrixes were calculated [27]. Non-metric multidimensional scaling (NMDS) analysis was performed to explore the similarity patterns among water or sediment samples based on the Bray-Curtis dissimilarity matrixes, by using the 'vegan' package in R. Canonical correspondence analysis (CCA) was employed to explore the relationship between bacterial community composition and environmental factors and to identify the physicochemical variables that may affect the temporal dynamics of the bacterial communities during the decomposition process. The community data matrix used in CCA analysis was based on the relative abundance of the abundant genera. In order to investigate the effects of environmental factors on the abundant genera, only the genera with average relative abundance > 1% and > 0.5% for water and sediment samples, respectively, were selected. Pearson correlation coefficients were calculated to identify the associations between environment variables and relative abundance of bacterial phyla/subphyla or bacterial richness/diversity for water samples. Only the phyla/subphyla with relative abundance > 1% were included in the correlation analysis.

## Results

### Changes of environmental factors during the *Microcystis* biomass decomposition process

**Fig 1:**
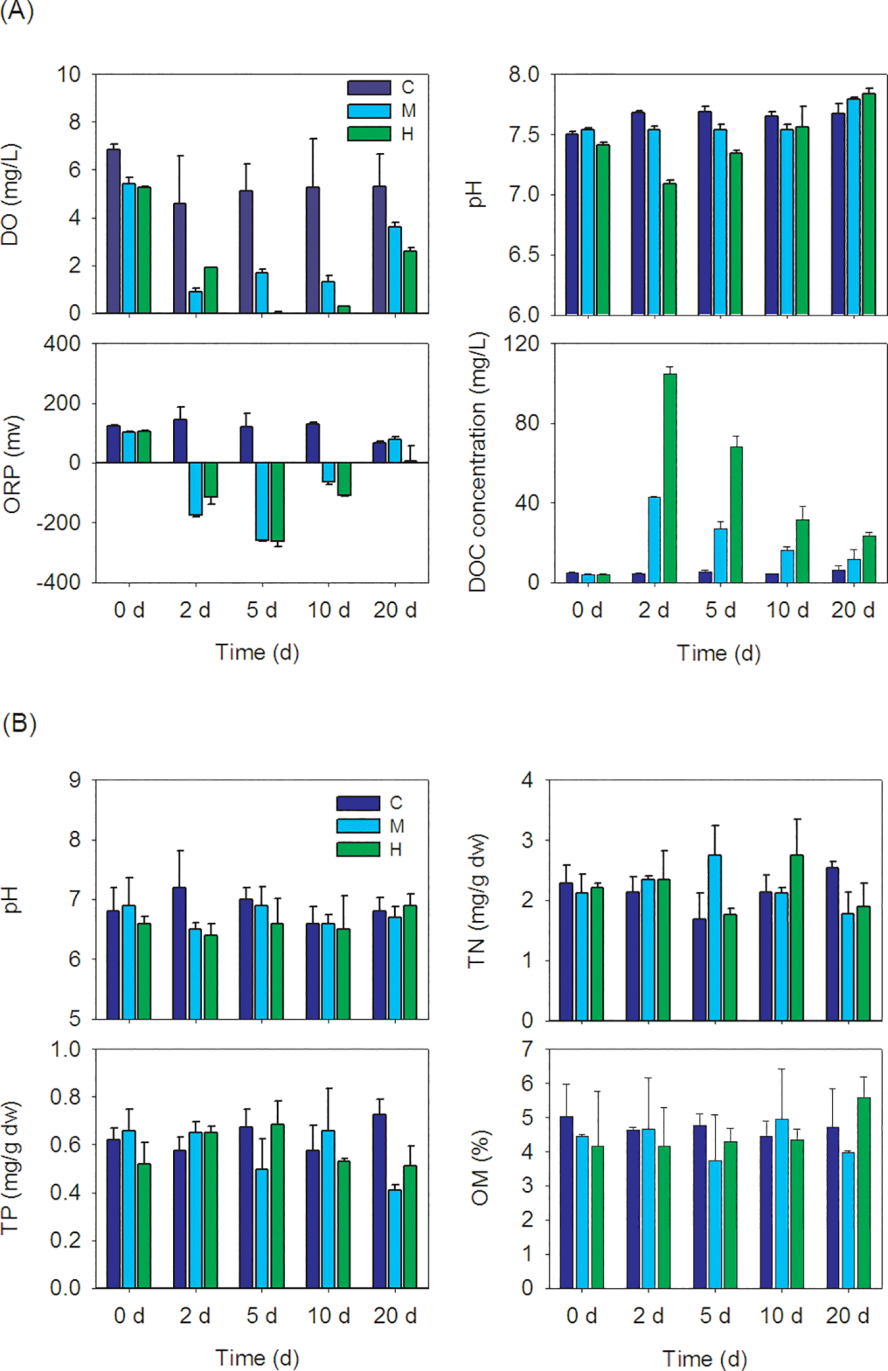
Variation of environmental factors in (A) water and (B) sediments of different treatments. C, control treatment without addition of *Microcystis*; M, moderate *Microcystis* biomass treatment; H, High *Microcystis* biomass treatment; DO, dissolved oxygen; ORP, oxidation reduction potential; DOC, dissolved organic carbon. TP, total phosphorus; TN, total nitrogen; OM, organic matter.

*Microcystis* biomass decomposed with sharp environmental changes in the water of the incubation systems. As shown in Fig 1A, for the H and M treatments, environmental factors such as pH, DO, ORP, and DOC of water samples always showed drastic changes during the *Microcystis* biomass decomposing process. The measured chemical factors of H treatment always showed extreme values on day 2 or day 5 and had higher concentrations of DOC and lower pH, DO, and ORP in water compared with the control group at the initial decomposition days (Fig 1A). In contrast, environmental factors of pH, total nitrogen (TN), total phosphorus (TP), and organic matter (OM) concentrations in the sediment samples did not show obvious variation during the *Microcystis* biomass decomposing process (Fig 1B).

### Variation of the bacterial taxon in water and sediments during the decomposition process

There were totally 84,382 16S rRNA gene sequences with mean length of 470 bp obtained from the 16 samples. The average numbers of the obtained sequences were about 2400 and 9000 for water and sediment samples, with the average coverage of 90% and 84%, respectively. The relative abundances of all detected bacterial phyla/subphyla in water and sediment samples were shown in S1 and S2 Tables. For water samples, *Betaproteobacteria* (relative abundance of 44.21% on average), *Alphaproteobacteria* (17.15%), and *Firmicutes* (10.77%) were the dominant bacterial groups, followed by *Bacteroidetes* (8.65%), *Gammaproteobacteria* (2.99%), *Epsilonproteobacteria* (1.97%), *Spirochaetes* (1.82%), and *Actinobacteria* (1.93%) (S1 Table). Other phyla such as *Acidobacteria* and *Chloroflexi* were present at relatively low abundances (< 1%) (S1 Table). Bacterial taxon in sediment samples showed considerably different patterns in comparison with water samples. As shown in S2 Table, *Chloroflexi* (16.82%), *Firmicutes* (15.45%) and *Actinobacteria* (11.60%) were abundant bacterial groups in sediments, followed by *Betaproteobacteria* (8.50%), *Gammaproteobacteria* (7.25%), *Acidobacteria* (5.07%), *Bacteroidetes* (4.07%), *Deltaproteobacteria* (3.70%), and *Alphaproteobacteria* (1.67%). The water and sediments contained distinct predominant bacterial groups (S1 and S2 Tables). *Betaproteobacteria* and *Alphaproteobacteria* were obviously abundant, whereas *Chloroflexi*, *Actinobacteria*, and *Acidobacteria* were rare in water in comparison with those in the sediments. Among the abundant phyla, only *Firmicutes* affiliated sequences were dominant (> 10% in average of the relative abundance) in both water and sediment samples.

**Fig 2:**
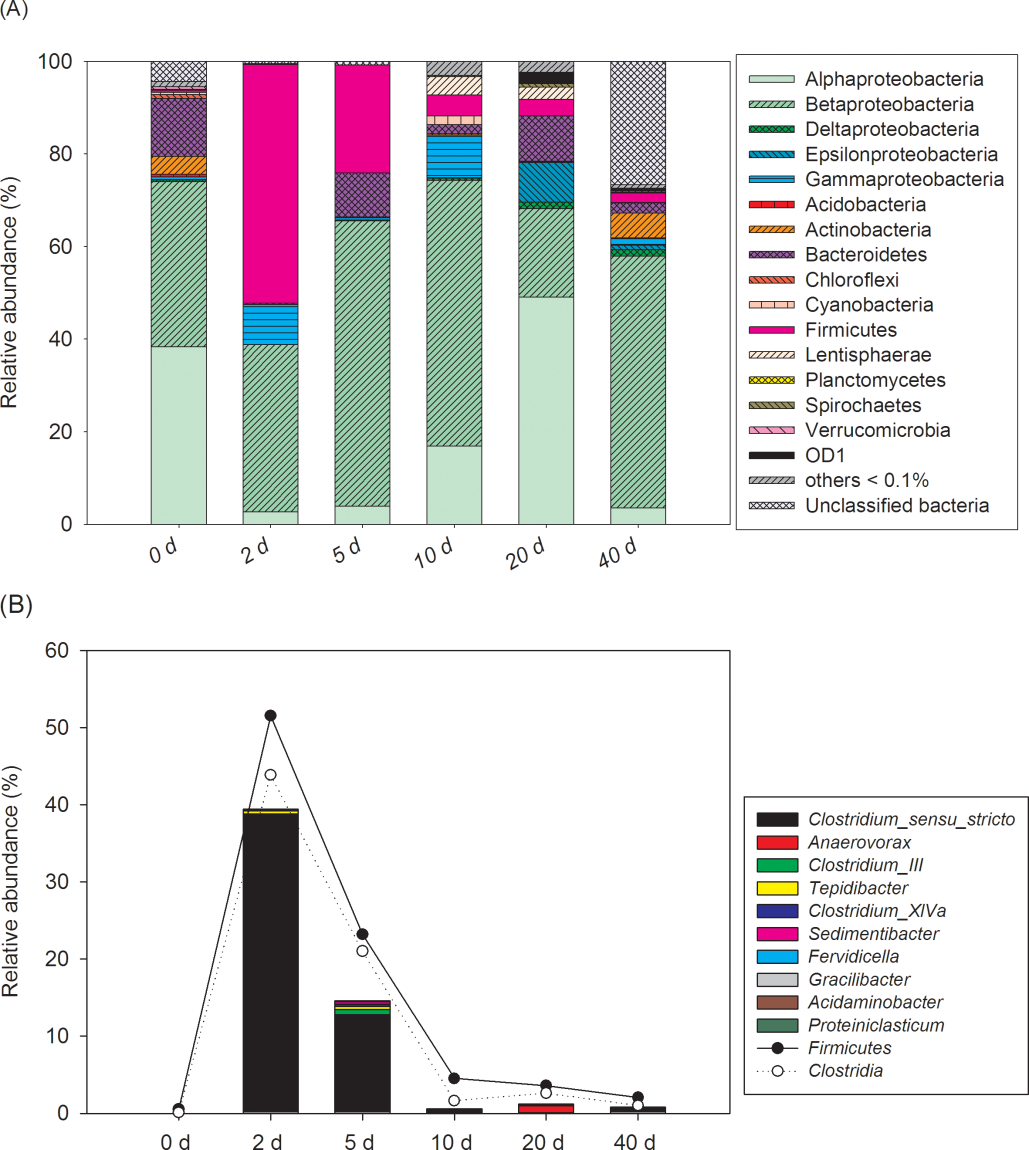
Relative abundance of (A) the dominant bacterial phyla/subphyla and (B) the top 10 genera in the phylum *Firmicutes* in water of the high *Microcystis* biomass treatment groups.

**Fig 3:**
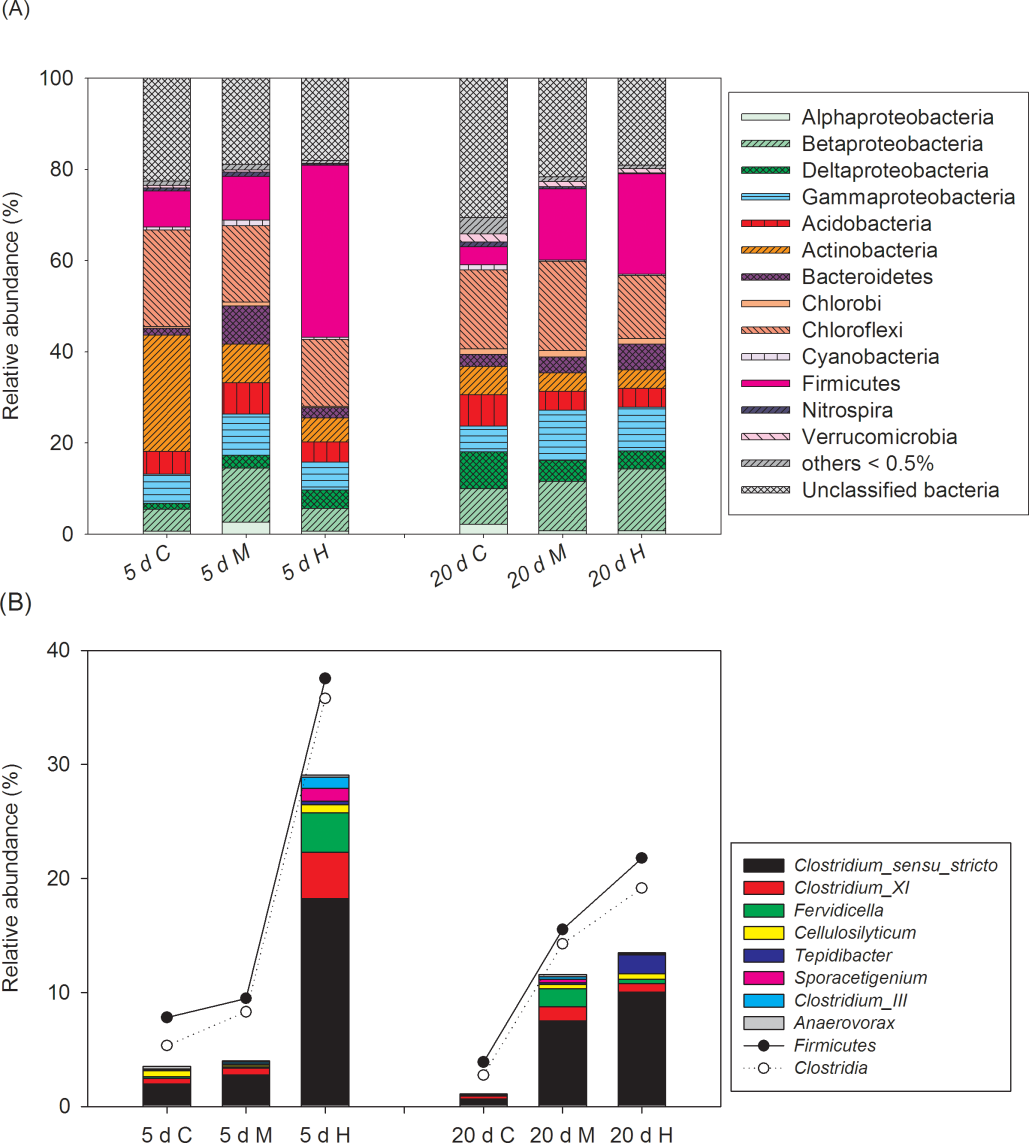
Relative abundance of (A) the dominant bacterial phyla/subphyla and (B) the top 10 genera in the phylum *Firmicutes* in the sediments of different treatments. C, control treatment without addition of *Microcystis*; M, moderate *Microcystis* biomass treatment; H, High *Microcystis* biomass treatment.

We found that the dominated bacterial groups showed different trends along the incubation time or in different *Microcystis* biomass addition treatments (Figs 2 and 3, S1 and S2 Tables). For water samples in H treatment, the dominant phyla (e.g., *Betaproteobacteria*, *Alphaproteobacteria*, and *Bacteroidetes*) showed no discernable trends, whereas the *Firmicutes* showed dramatic variation with incubation time during the decomposition process (Fig 2A). As shown in Fig 2B, the relative abundance of *Firmicutes* increased from 0.54% at the beginning of the experiment to peak value (51.61%) on day 2 and decreased to 3.60% on day 20. Within the *Firmicutes* phylum, the genus *Clostridium sensu stricto* (*Clostridia* class, *Clostridiales* order, *Clostridiaceae* 1 family) was the absolutely dominant group, which comprised 75.27% of *Firmicutes* sequences on day 2 for water samples. The relative abundance of *Clostridia* class or *Clostridium sensu stricto* genus showed similar variation trends as *Firmicutes* during the decomposition process and peak values were also observed on day 2. For different *Microcystis* addition treatments (S1 Table), the relative abundance of *Firmicutes* of H treatment (23.29%) was greater than those of the M treatment (9.41%) on day 5 for water samples. After incubation for 20 days, the gaps of the values between different treatments became smaller.

For sediment samples, the relative abundance of *Firmicutes* also showed obvious variation with incubation time during the decomposition process (S2 Table). Similar as water samples, the *Firmicutes* in sediments showed peak value on day 5 (37.81% in relative abundance) and then decreased to 21.97% on day 20. The genus *Clostridium sensu stricto* was also dominant member in *Firmicutes*, which comprised 48.58% of *Firmicutes* sequences on day 5. For different *Microcystis* addition treatments (Fig 3A), despite some dominant phyla (e.g., subphyla of *Proteobacteria*, *Chloroflexi*, and *Acidobacteria*) showed no discernable trends, the relative abundance of *Firmicutes* in H treatments was greater than M and C treatments on day 5 and day 20. The dominant class *Clostridia* or genus *Clostridium sensu stricto* showed similar variation trends as *Firmicutes* in different treatment groups (Fig 3B).

Based on the relative abundance of genera, the water and sediments contained distinct predominant genera (S1 and S2 Figs). For water samples, the genera such as *Malikia* (affiliated with *Betaproteobacteria*), *Clostridium sensu stricto* (*Firmicutes*), *Azospirillum* (*Alphaproteobacteria*), *Polynucleobacter* (*Betaproteobacteria*), *Vogesella* (*Betaproteobacteria*), and *Acidovorax* (*Betaproteobacteria*) showed dramatically variation during the decomposing process and the relative abundance was greater than 10% in some water samples (S1 Fig). In contrast, the relative abundance was almost below 5% for all genera except *Clostridium sensu stricto* in the sediments (S2 Fig). The relative abundances of *Clostridium sensu stricto* in *Microcystis* biomass addition treatments were always greater than those in the control treatment (S1 and S2 Figs).

### Bacterial diversity and community composition in water and sediments

For water samples, the number of OTUs in the H treatment was lower in comparison with the control treatment (Table 1). This result was also confirmed by the Faith’s PD diversity index, which declined after *Microcystis* addition and was lower in comparison with the control treatment. The M treatment also showed lower bacterial diversity compared with the control treatment on day 5 and day 20. For the sediment samples, the diversity indices showed no obvious variations after *Microcystis* addition (Table 1).

**Table 1:** Bacterial richness and diversity of the bacterial community in water and sediments of different treatments.

Samples	OTUs	Shannon	Faith’s PD
**Water**			
0 d C	485	4.36	32.28
20 d C	424	5.08	32.26
5 d M	386	4.70	24.36
20 d M	214	3.84	16.52
2 d H	326	4.34	17.51
5 d H	390	4.16	21.49
10 d H	239	3.64	16.47
20 d H	288	3.86	19.86
40 d H	248	3.04	19.76
**Sediments**			
5 d C	1839	6.59	114.69
20 d C	1904	6.68	120.29
5 d M	2850	7.45	188.26
20 d M	2085	6.78	133.73
2 d H	1731	6.50	112.03
5 d H	1767	6.44	110.14
20 d H	1892	6.46	116.57

Operational taxonomic units, OTUs; Shannon index, Shannon; the Faith’s phylogenetic diversity, Faith’s PD. C, control treatment without addition of *Microcystis*; M, moderate *Microcystis* biomass treatment; H, High *Microcystis* biomass treatment.

**Fig 4:**
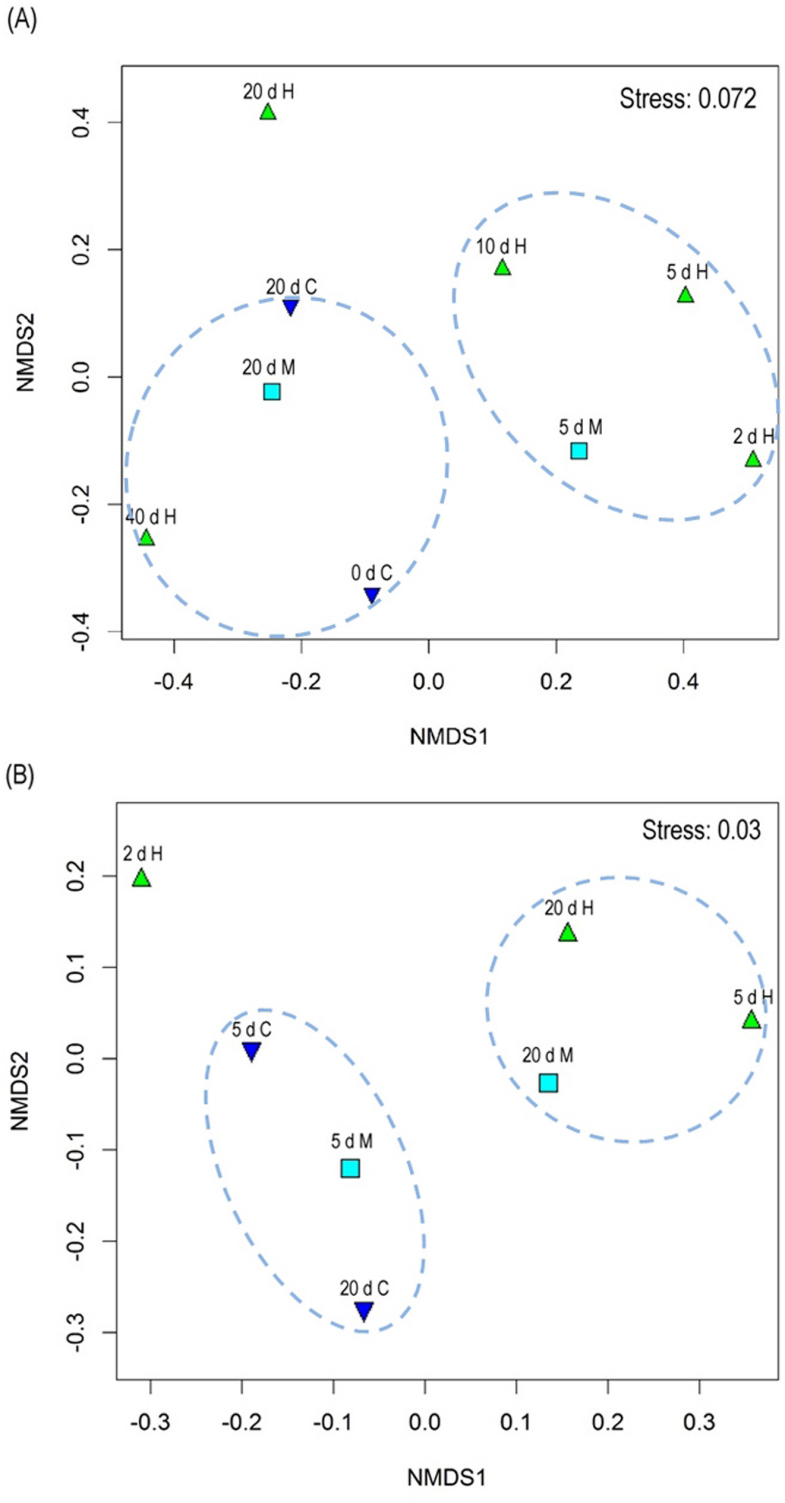
Non-metric multidimensional scaling (NMDS) analysis of (A) water and (B) sediment samples. C, control treatment without addition of *Microcystis*; M, moderate *Microcystis* biomass treatment; H, High *Microcystis* biomass treatment.

Non-metric multidimensional scaling (NMDS) analysis was performed to characterize the similarity of bacterial communities in water and sediments (Fig 4). Both the *Microcystis* addition and incubation time remarkably affected the bacterial community similarity among samples. For water samples, bacterial communities of the H treatment (2 d H, 5 d H, and 10 d H) and M treatment (5 d M) in the earlier decomposition stage could be clustered into one group, whereas the bacterial communities of the H and M treatments in the post decomposition stage (40 d H and 20 d M) and control treatment (0 d C and 20 d C) were quite near to each other (Fig 4A). For sediment samples, bacterial communities of the H treatment could be clearly separated from the control treatment (Fig 4B).

### Relationships between environmental factors and bacterial communities

For water samples, some abundant bacterial phyla showed significant correlations with environmental factors (Table 2). For example, the relative abundance of *Firmicutes* showed strong positive correlations with DOC and turbidity (*P* < 0.01) and significant negative correlations with pH (*P* < 0.01) and DO (*P* < 0.05). The relative abundance of *Alphaproteobacteria*, *Bacteroidetes*, and *Epsilonproteobacteria* were significantly related to pH, DOC, and TN, respectively (*P* < 0.05). For sediment samples, no significant correlations were found between the relative abundance of bacterial phyla and environmental factors (*P* > 0.05 in all cases).

**Table 2:** Pearson correlation coefficients between the environmental variables and relative abundance of bacterial phyla/subphyla for water samples.

Phyla/subphyla	pH	DO	DOC	Turbidity	ORP	TP	TN
*Betaproteobacteria*	-0.337	-0.504	0.200	0.256	-0.655	-0.380	-0.262
*Alphaproteobacteria*	0.784*	0.598	-0.571	-0.705	0.441	-0.120	0.468
*Firmicutes*	-0.864**	-0.715*	0.972***	0.998***	-0.335	0.225	-0.239
*Bacteroidetes*	0.444	0.650	-0.731*	-0.659	0.292	-0.325	0.077
*Gammaproteobacteria*	-0.465	-0.444	0.496	0.471	-0.058	0.318	-0.373
*Epsilonproteobacteria*	0.690	0.319	-0.395	-0.444	0.399	0.517	0.825*
*Actinobacteria*	0.114	0.678	-0.614	-0.530	0.420	-0.674	-0.524
*Spirochaetes*	0.134	0.293	-0.369	-0.427	0.458	0.313	0.385

Only the phyla/subphyla with relative abundance > 1% were included in the analysis. DO, dissolved oxygen; DOC, dissolved organic carbon; ORP, oxidation reduction potential; TP, total phosphorus; TN, total nitrogen. Coefficient values in bold indicate significant correlation at *P* < 0.05 level.

* *P* < 0.05

** *P* < 0.01

*** *P* < 0.001.

**Fig 5:**
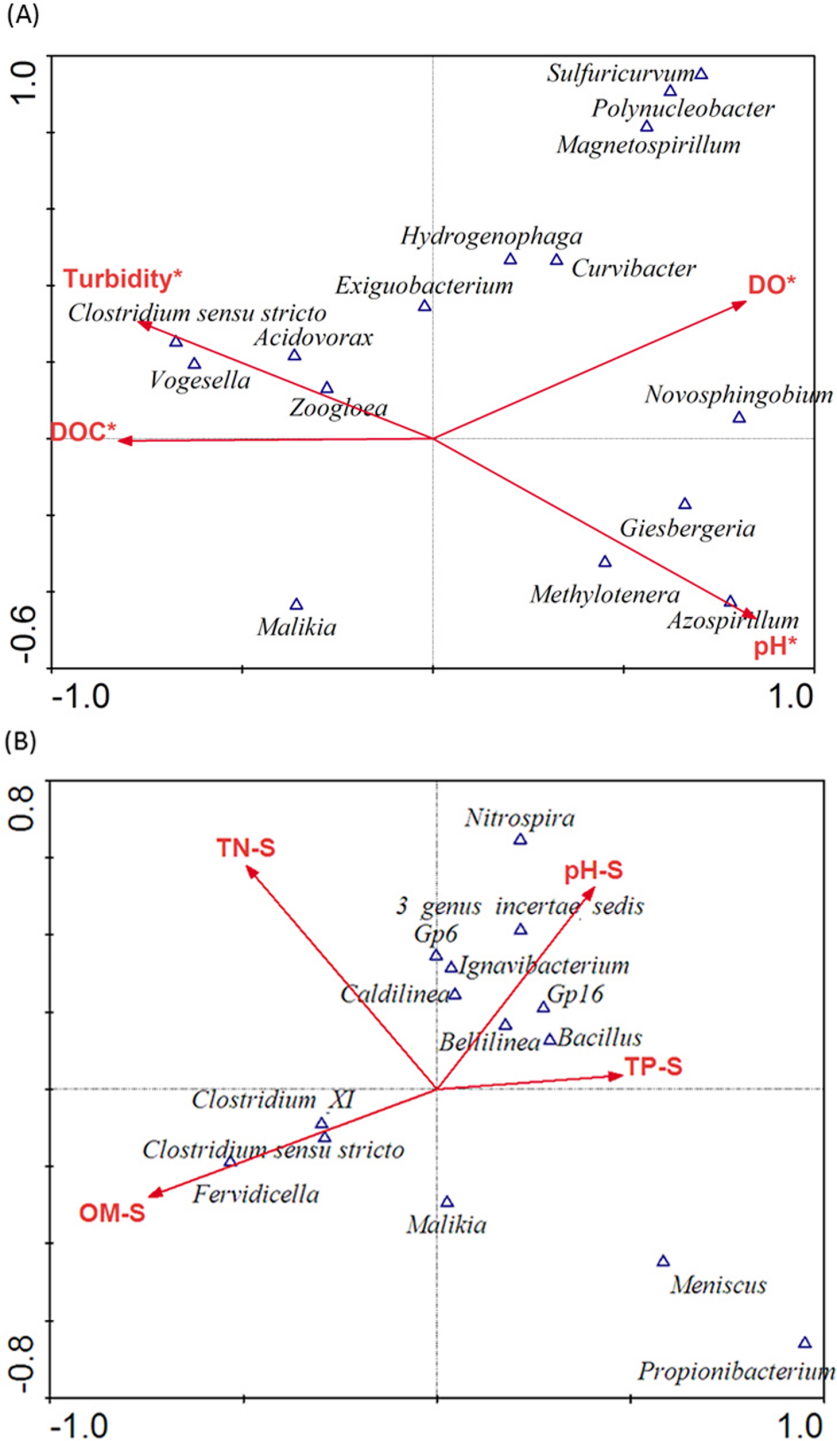
Ordination biplots of canonical correspondence analysis (CCA) of environmental variables and bacterial genera in (A) water and (B) sediments of the incubation systems. The relative abundances of the involved genera are > 1% and > 0.5% for water and sediment samples on average. DO, dissolved oxygen; DOC, dissolved organic carbon; ORP, oxidation reduction potential; TP, total phosphorus; TN, total nitrogen; OM, organic matter; -S, Environmental variables in the sediments. * *P* < 0.05, Monte Carlo permutation test.

To further investigate the relationship between bacterial community composition and environmental factors, canonical correspondence analysis (CCA) was performed. For water samples, the pH, DO, DOC, and turbidity showed significant effects on the relative abundance of the dominant bacterial genera in water samples and accounted for the major variations (44. 6%) in the bacterial composition (*P* < 0.05) (Fig 5A, Table 3). The other examined environmental variables, including ORP, TN, and TP, did not exhibit significant relationship with the bacterial community composition (*P* > 0.05). As shown in Fig 5A, different bacteria genera appeared to adapt to different environmental conditions. Especially, the abundant genus, *Clostridium sensu stricto*, was positively correlated with the DOC and turbidity and negatively correlated with pH and DO.

**Table 3:** Results of canonical correspondence analysis (CCA) of environmental variables and bacterial genera (relative abundance >1% on average) for water and sediment samples.

**Environmental variables**		**pH**	**DO**	**DOC**	**Turbidity**	**ORP**	**TP**	**TN**
**Bacterial genera in water**	*F*	**2.620**	**2.381**	**2.411**	**2.283**	1.618	1.307	1.792
	*P*	**0.002**	**0.012**	**0.006**	**0.004**	0.162	0.286	0.054
		**pH-S**	**OM-S**	**TP-S**	**TN-S**			
**Bacterial genera in the sediments**	*F*	2.220	1.830	1.630	1.720			
	*P*	0.162	0.182	0.274	0.226			

DO, dissolved oxygen; DOC, dissolved organic carbon; ORP, oxidation reduction potential; TP, total phosphorus; TN, total nitrogen; OM, organic matter; -S, Environmental variables in the sediments. Coefficient values in bold indicate significant correlations at *P* < 0.05 level.

For the sediment samples, bacterial genera and chemical variables including pH, TN, TP, and OM were involved in CCA analysis. Even though the chosen sediment chemical variables did not significantly explain the variations in bacterial genera in the present study (*P* > 0.05 in all cases, Table 3), it can be observed in Fig 5B that the genera *Clostridium sensu stricto* were positively correlated with OM and negatively correlated with pH in the sediments, which corresponded with the results of water sample analysis.

## Discussion

### The genus *Clostridium sensu stricto* involved in *Microcystis* decomposition process

In the present study, *Microcystis* decomposition had dramatic influence on the bacterial taxon especially for the genus *Clostridium sensu stricto* (*Firmicutes* phylum, *Clostridia* class, *Clostridiales* order, *Clostridiaceae* 1 family) in water and sediment samples. The *Clostridium sensu stricto* was the most abundant member within *Firmicutes* and showed great variation during the *Microcystis* decomposing process. In previous anaerobic incubation experiments, Wu et al. [28] isolated two strains of anaerobic bacteria affiliated to *Clostridium* from decomposing algal scums in Lake Taihu. Xing et al. [10] examined the bacteria involved in the anaerobic decomposition of *Microcystis* biomass and found that *Clostridium* clusters and their diverse consortiums dominated the bacterial communities during the anaerobic decomposition process in lake water.

It is suggested that the abundant *Firmicutes*, in particular the *Clostridium* members, may be responsible for the decomposition of *Microcystis* substrates. The taxonomic information for the genus *Clostridium* has not been reported in detail [29–30]. Based on the previous description, species of the genus *Clostridium* have common characteristics, such as being strictly anaerobic, spore-forming, Gram-stain positive, having low G+C contents and producing both acid and alcohol during fermentation [28]. This group of anaerobes is not very homogeneous, and which includes microorganisms covering a wide ecological and physiological range and can be found in many extreme environments [10, 30]. The members of the *Clostridium* are metabolically versatile to degrade different kinds of organic matters as well as of the high rate of the breakdown processes when they take place under optimal conditions [30–34].

In the present study, the relative abundances of the genus *Clostridium sensu stricto* were quite low in the control treatment, but showed dramatic increasing after *Microcystis* biomass addition. For different *Microcystis* addition treatments, the relative abundances of *Clostridium sensu stricto* were high *Microcystis* biomass treatment > moderate *Microcystis* biomass treatment > control treatment in the initial decomposing periods. Among all the genera, only *Clostridium sensu stricto* was dominant (> 6% in average relative abundance) in both water and sediment samples in the *Microcystis* addition systems. These results suggested that the genus *Clostridium sensu stricto* might play a dominant role in *Microcystis* biomass decomposing in aquatic ecosystems.

### Dissimilarity and diversity of bacterial communities influenced by *Microcystis* decomposition

Based on the results of the NMDS analysis, it was found that both *Microcystis* addition and incubation time could exhibit remarkable influence on the bacterial community similarities among samples. For water samples, bacterial communities of the *Microcystis* biomass addition treatments in the earlier decomposition stage could be clustered into one group, whereas the bacterial communities of the *Microcystis* addition treatments in the post decomposition stage were quite similar to the control treatment, suggesting that bacterial communities could recover after 20 d or 40 d incubation time for the moderate or high *Microcystis* addition treatments. For sediment samples, bacterial communities of the high *Microcystis* biomass treatment could also be clearly separated from the control treatment.

Several previous studies have examined the bacterial community composition dynamics in response to the breakdown of *Microcysti*s biomass in freshwater lakes [9, 11, 18]. For example, Li et al. [9] found that the breakdown of *Microcystis* biomass had strong impacts on bacterioplankton community composition and some pathogens such as *Micrococcineae* might associate with the decomposition of *Microcystis* biomass. Shao et al. [11, 18] reported that the composition of the particle-attached or sediment bacterial communities varied temporally during the decomposition of *Microcystis* biomass. These previous studies found that *Microcystis* decomposition did drive the shift in bacterial communities in water or sediments. However, limited sampling time points were selected in these previous studies and the variation dynamics of bacterial community composition were not thoroughly illustrated.

The diversity and structure of the bacterial communities have been reported to show remarkable changes during the biodegradation or decomposition process [13, 35–36]. Dilly et al. [35] found that the diversity indices such as the Shannon-Weaver index, evenness, and equitability increased during the course of decomposition. Das et al. [36] found that the bacterial richness increased in the earlier decomposition stage and reduced at the post decomposition stage. In the present study, the *Microcystis* addition treatments always showed lower bacterial richness and diversity than did the control treatment in water. As suggested in previous studies, extreme disturbances would decrease compositional stochasticity by acting as selection factors [37]. It was supposed that *Microcystis* addition could execute extreme disturbances on the bacterial communities. For sediment samples, the diversity indices showed no obvious variation during the decomposing process for different treatments, which may be due to the higher bacterial diversity in the sediments.

### Relationships between environmental factors and bacterial communities

The decomposition of *Microcystis* blooms always results in drastic environmental changes. In the present study, dramatic increase in DOC and drops in pH, DO, and ORP were observed in water, which was similar as previous studies [11, 15]. Environmental variation played important roles in structuring the microbial assemblages [38]. As described in previous studies, factors, such as the ecological role of the taxa (generalists versus specialists), stage of decay, and time of exposure, appeared to be important determinants of microbial community structure [36]. In the present study, the dramatic physicochemical changes induced by decomposition of *Microsystis* biomass may have great impacts on the bacterial communities. The results of CCA analysis demonstrated that the pH, DO, DOC, and turbidity showed significant association with the relative abundance of the dominant bacterial genera in water samples. Some bacterial groups (i.e., the genus *Clostridium sensu stricto*) showed significant positive or negative correlations with the environmental factors.

Following were possible explanations for the observed variations in bacterial community composition during the decomposition process. On one hand, the variations of environmental conditions (i.e., higher DOC, and lower pH, DO, and ORP values), which were induced by the breakdown of *Microcystis* biomass, might act as niche selection factors and be prominent forces driving the variation of bacterial community composition during the decomposing process. On the other hand, the dominant bacterial groups might be well adapt to the shifted environmental conditions and participate in *Microcystis* decomposition process [16]. Previous studies have demonstrated that *Clostridium* significantly correlated with anaerobic decomposition of organic matter and well grew under lower pH conditions [10, 28, 39]. In the present study, the *Microcystis* derived high DOC and low DO concentrations were thought to be very important in maintaining the abundant genus, *Clostridium sensu stricto*. Even though no significant correlations between sediment chemical variables and bacterial groups were observed, positive correlation with OM and negative correlation with pH were observed for the genus *Clostridium* in the sediments in CCA analysis, which corresponded well with the observations for water samples.

## Conclusions

In conclusion, the results of this study demonstrated that *Microcystis* decomposition had drastic influence on bacterial community composition. *Firmicutes* were dominant in both water and sediments. The genus *Clostridium sensu stricto* was the absolutely dominant member and showed drastic variation with incubation time during the decomposition process. Environmental factors in water (e.g., pH, DO, and DOC) showed dramatic variation during the decomposing process, which might be the prominent forces driving the variation of bacterial communities. The abundant genus, *Clostridium sensu stricto*, were thought to be well adapted to higher DOC and turbidity and lower pH and DO conditions.

## Supporting information

S1 Fig Relative abundance of the top 10 genera in each water sample of different treatments. C, control treatment without addition of *Microcystis*; M, moderate *Microcystis* biomass treatment; H, High *Microcystis* biomass treatment.
(DOCX)

S2 Fig Relative abundance of the top 10 genera in each sediments sample of different treatments. C, control treatment without addition of *Microcystis*; M, moderate *Microcystis* biomass treatment; H, High *Microcystis* biomass treatment.
(DOCX)

S1 Table Relative abundance of all detected bacterial phyla/subphyla in water of different treatments. (DOCX)

S2 Table Relative abundance of all detected bacterial phyla/subphyla in sediments of different treatments. (DOCX)

## References

[1] Eiler A, Bertilsson S. Composition of freshwater bacterial communities associated with cyanobacterial blooms in four Swedish lakes. Environ Microbiol. 2004; 6: 1228–1243. doi: 10.1111/j.1462-2920.2004.00657.x 15560821 10.1111/j.1462-2920.2004.00657.x

[2] Buchan A, LeCleir GR, Gulvik CA, González JM. Master recyclers: features and functions of bacteria associated with phytoplankton blooms. Nat Rev Microbiol. 2014; 12: 686–698. doi: 10.1038/nrmicro3326 25134618 10.1038/nrmicro3326

[3] Chen MJ, Chen FZ, Xing P, Li HB, Wu QL. Microbial eukaryotic community in response to *Microcystis* spp. bloom, as assessed by an enclosure experiment in Lake Taihu, China. FEMS Microbiol Ecol. 2010; 74: 19–31. doi: 10.1111/j.1574-6941.2010.00923.x 20597984 10.1111/j.1574-6941.2010.00923.x

[4] Guo L. Doing battle with the green monster of Taihu Lake. Science. 2007; 317: 1166 doi: 10.1126/science.317.5842.1166 17761862 10.1126/science.317.5842.1166

[5] Zhang Z, Zhang XX, Wu B, Yin J, Yu Y, Yang L. Comprehensive insights into microcystin-LR effects on hepatic lipid metabolism using cross-omics technologies. J Hazard Mater. 2016; 315: 126–134. doi: 10.1016/j.jhazmat.2016.05.011 27208774 10.1016/j.jhazmat.2016.05.011

[6] Grossart H, Czub G, Simon M. Algae-bacteria interactions and their effects on aggregation and organic matter flux in the sea. Environ Microbiol. 2006; 8: 1074–1084. doi: 10.1111/j.1462-2920.2006.00999.x 16689728 10.1111/j.1462-2920.2006.00999.x

[7] Landa M, Blain S, Christaki U, Monchy S, Obernosterer I. Shifts in bacterial community composition associated with increased carbon cycling in a mosaic of phytoplankton blooms. ISME J. 2015; 10: 39–50. doi: 10.1038/ismej.2015.105 26196334 10.1038/ismej.2015.105 PMC4681851

[8] Parveen B, Ravet V, Djediat C, Mary I, Quiblier C, Debroas D, et al Bacterial communities associated with *Microcystis* colonies differ from free-living communities living in the same ecosystem. Environ Microbiol Rep. 2013; 5: 716–724. doi: 10.1111/1758-2229.12071 24115622 10.1111/1758-2229.12071

[9] Li HB, Xing P, Wu QL. Characterization of the bacterial community composition in a hypoxic zone induced by *Microcystis* blooms in Lake Taihu, China. FEMS Microbiol Ecol. 2012; 79: 773–784. doi: 10.1111/j.1574-6941.2011.01262.x 22126440 10.1111/j.1574-6941.2011.01262.x

[10] Xing P, Guo L, Tian W, Wu QL. Novel *Clostridium* populations involved in the anaerobic degradation of *Microcystis* blooms. ISME J. 2011; 5: 792–800. doi: 10.1038/ismej.2010.176 21107445 10.1038/ismej.2010.176 PMC3105768

[11] Shao KQ, Zhang L, Wang YP, Yao X, Tang XM, Qin BQ, et al The responses of the taxa composition of particle-attached bacterial community to the decomposition of *Microcystis* blooms. Sci Total Environ. 2014; 488: 236–242. doi: 10.1016/j.scitotenv.2014.04.101 24836132 10.1016/j.scitotenv.2014.04.101

[12] Qin BQ, Xu PZ, Wu QL, Luo LC, Zhang YL. Environmental issues of Lake Taihu, China. Hydrobiologia. 2007; 581: 3–14.

[13] Wang SG, Diao XJ, He LS. Effects of algal bloom formation, outbreak, and extinction on heavy metal fractionation in the surficial sediments of Chaohu Lake. Environ Sci Pollut Res. 2015; 22: 14269–14279. 10.1007/s11356-015-4652-y 25976327

[14] Xie LQ, Xie P, Tang HJ. Enhancement of dissolved phosphorus release from sediment to lake water by *Microcystis* blooms-an enclosure experiment in a hyper-eutrophic, subtropical Chinese lake. Environ Pollut. 2003; 122: 391–399. 12547528 10.1016/s0269-7491(02)00305-6

[15] Zhu MY, Zhu GW, Zhao LL, Yao X, Zhang YL, Gao G, et al Influence of algal bloom degradation on nutrient release at the sediment-water interface in Lake Taihu, China. Environ Sci Pollut Res. 2013; 20: 1803–1811. 10.1007/s11356-012-1084-9 22825639

[16] Strickland MS, Lauber C, Fierer N, Bradford MA. Testing the functional significance of microbial community composition. Ecology. 2009; 90: 441–451. 19323228 10.1890/08-0296.1

[17] Leff JW, Nemergut DR, Grandy AS, O’Neill SP, Wickings K, Townsend AR, et al The effects of soil bacterial community structure on decomposition in a tropical rain forest. Ecosystems. 2012; 15: 284–298.

[18] Shao K, Gao G, Chi K, Qin B, Tang X, Yao X, et al Decomposition of *Microcystis* blooms: Implications for the structure of the sediment bacterial community, as assessed by a mesocosm experiment in Lake Taihu, China. J Basic Microb. 2013; 53: 549–554. 10.1002/jobm.201100532 22915041

[19] Chen Y, Qin B, Teubner K, Dokulil MT. Long-term dynamics of phytoplankton assemblages: *Microcystis*-domination in Lake Taihu, a large shallow lake in China. J Plankton Res. 2003; 25: 445–453.

[20] Shi XL, Kong FX, Yu Y, Yang Z. Survival of *Microcystis aeruginosa* and *Scenedesmus obliquus* under dark anaerobic conditions. Mar Freshwater Res. 2007; 58: 634–639.

[21] Jin XC, Tu QY. Survey specification for lake eutrophication. 2nd ed. Environmental Science Press, Beijing, China; 1990.

[22] Zeng J, Bian YQ, Xing P, Wu QL. Macrophyte species drive the variation of bacterioplankton community composition in a shallow freshwater lake. Appl Environ Microbiol. 2012; 78: 177–184. doi: 10.1128/AEM.05117-11 22038598 10.1128/AEM.05117-11 PMC3255635

[23] Zhao DY, Huang R, Zeng J, Yu ZB, Liu P, Cheng SP, et al Pyrosequencing analysis of bacterial community and assembly in activated sludge samples from different geographic regions in China. Appl Microbiol Biotechnol. 2014; 98: 9119–9128. doi: 10.1007/s00253-014-5920-3 25022664 10.1007/s00253-014-5920-3

[24] Zeng J, Zhao DY, Li HB, Huang R, Wang JJ, Wu QL. A monotonically declining elevational pattern of bacterial diversity in freshwater lake sediments. Environ Microbiol. 2016; 18: 5175–5186. doi: 10.1111/1462-2920.13526 27632715 10.1111/1462-2920.13526

[25] Faith DP. Conservation evaluation and phylogenetic diversity. Biol Conserv. 1992; 61: 1–10.

[26] Hill TCJ, Walsh KA, Harris JA, Moffett BF. Using ecological diversity measures with bacterial communities. FEMS Microbiol Ecol. 2003; 43: 1–11. doi: 10.1111/j.1574-6941.2003.tb01040.x 19719691 10.1111/j.1574-6941.2003.tb01040.x

[27] Lozupone C, Hamady M, Knight R. UniFrac–an online tool for comparing microbial community diversity in a phylogenetic context. BMC Bioinformatics. 2006; 7: 371 doi: 10.1186/1471-2105-7-371 16893466 10.1186/1471-2105-7-371 PMC1564154

[28] Wu YF, Zheng H, Wu QL, Yang H, Liu SJ. *Clostridium algifaecis* sp. nov., an anaerobic bacterial species from decomposing algal scum. Int J Syst Evol Microbiol. 2014; 64: 3844–3848. doi: 10.1099/ijs.0.064345-0 25168611 10.1099/ijs.0.064345-0

[29] Gupta RS, Gao B. Phylogenomic analyses of clostridia and identification of novel protein signatures that are specific to the genus *Clostridium sensu stricto* (cluster I). Int J Syst Evol Microbiol. 2009; 59: 285–294. doi: 10.1099/ijs.0.001792-0 19196767 10.1099/ijs.0.001792-0

[30] Dickinson CH. Biology of plant litter decomposition. Elsevier, 2012.

[31] Burrell PC, O'Sullivan C, Song H, Clarke WP, Blackall LL. Identification, detection, and spatial resolution of *Clostridium* populations responsible for cellulose degradation in a methanogenic landfill leachate bioreactor. Appl Environ Microbiol. 2004; 70: 2414–2419. doi: 10.1128/AEM.70.4.2414-2419.2004 15066839 10.1128/AEM.70.4.2414-2419.2004 PMC383074

[32] Kato S, Haruta S, Cui ZJ, Ishii M, Igarashi Y. Effective cellulose degradation by a mixed-culture system composed of a cellulolytic *Clostridium* and aerobic non-cellulolytic bacteria. FEMS Microbiol Ecol. 2004; 51: 133–142. doi: 10.1016/j.femsec.2004.07.015 16329862 10.1016/j.femsec.2004.07.015

[33] Shiratori H, Sasaya K, Ohiwa H, Ikeno H, Ayame S, Kataoka N, et al *Clostridium clariflavum* sp. nov. and *Clostridium caenicola* sp. nov., moderately thermophilic, cellulose-/cellobiose-digesting bacteria isolated from methanogenic sludge. Int J Syst Evol Microbiol. 2009; 59: 1764–1770. doi: 10.1099/ijs.0.003483-0 19542130 10.1099/ijs.0.003483-0

[34] Herring CD, Thorne PG, Lynd LR. *Clostridium thermocellum* releases coumaric acid during degradation of untreated grasses by the action of an unknown enzyme. Appl Microbiol Biotechnol. 2016; 100: 2907–2915. doi: 10.1007/s00253-016-7294-1 26762388 10.1007/s00253-016-7294-1

[35] Dilly O, Bloem J, Vos A, Munch JC. Bacterial diversity in agricultural soils during litter decomposition. Appl Environ Microbiol. 2004; 70: 468–474. doi: 10.1128/AEM.70.1.468-474.2004 14711676 10.1128/AEM.70.1.468-474.2004 PMC321295

[36] Das M, Royer TV, Leff LG. Diversity of fungi, bacteria, and actinomycetes on leaves decomposing in a stream. Appl Environ Microbiol. 2007; 73: 756–767. doi: 10.1128/AEM.01170-06 17142366 10.1128/AEM.01170-06 PMC1800785

[37] Zhou J, Deng Y, Zhang P, Xue K, Liang Y, Van Nostrand JD et al Stochasticity, succession, and environmental perturbations in a fluidic ecosystem. Proc Natl Acad Sci. 2014; 111: E836–E845. doi: 10.1073/pnas.1324044111 24550501 10.1073/pnas.1324044111 PMC3948316

[38] Liu LM, Yang J, Yu XQ, Chen GJ, Yu Z. Patterns in the composition of microbial communities from a subtropical river: effects of environmental, spatial and temporal factors. PloS One. 2013; 8: e81232 doi: 10.1371/journal.pone.0081232 24244735 10.1371/journal.pone.0081232 PMC3828266

[39] Schoefer L, Mohan R, Schwiertz A, Braune A, Blaut M. Anaerobic decomposition of flavonoids by *Clostridium orbiscindens*. Appl Environ Microbiol. 2003; 69: 5849–5854. doi: 10.1128/AEM.69.10.5849-5854.2003 14532034 10.1128/AEM.69.10.5849-5854.2003 PMC201214

