# Supplementary material for: Variation of bacterial communities in water and sediments during the decomposition of Microcystis biomass
Source: PLoS One. 2017 Apr 24;12(4):e0176397. doi: 10.1371/journal.pone.0176397 (PMC5402945; doi:10.1371/journal.pone.0176397)
Supplement: S2 Fig — C, control treatment without addition of Microcystis; M, moderate Microcystis biomass treatment; H, High Microcystis biomass treatment. (DOCX) [file pone.0176397.s002.docx]

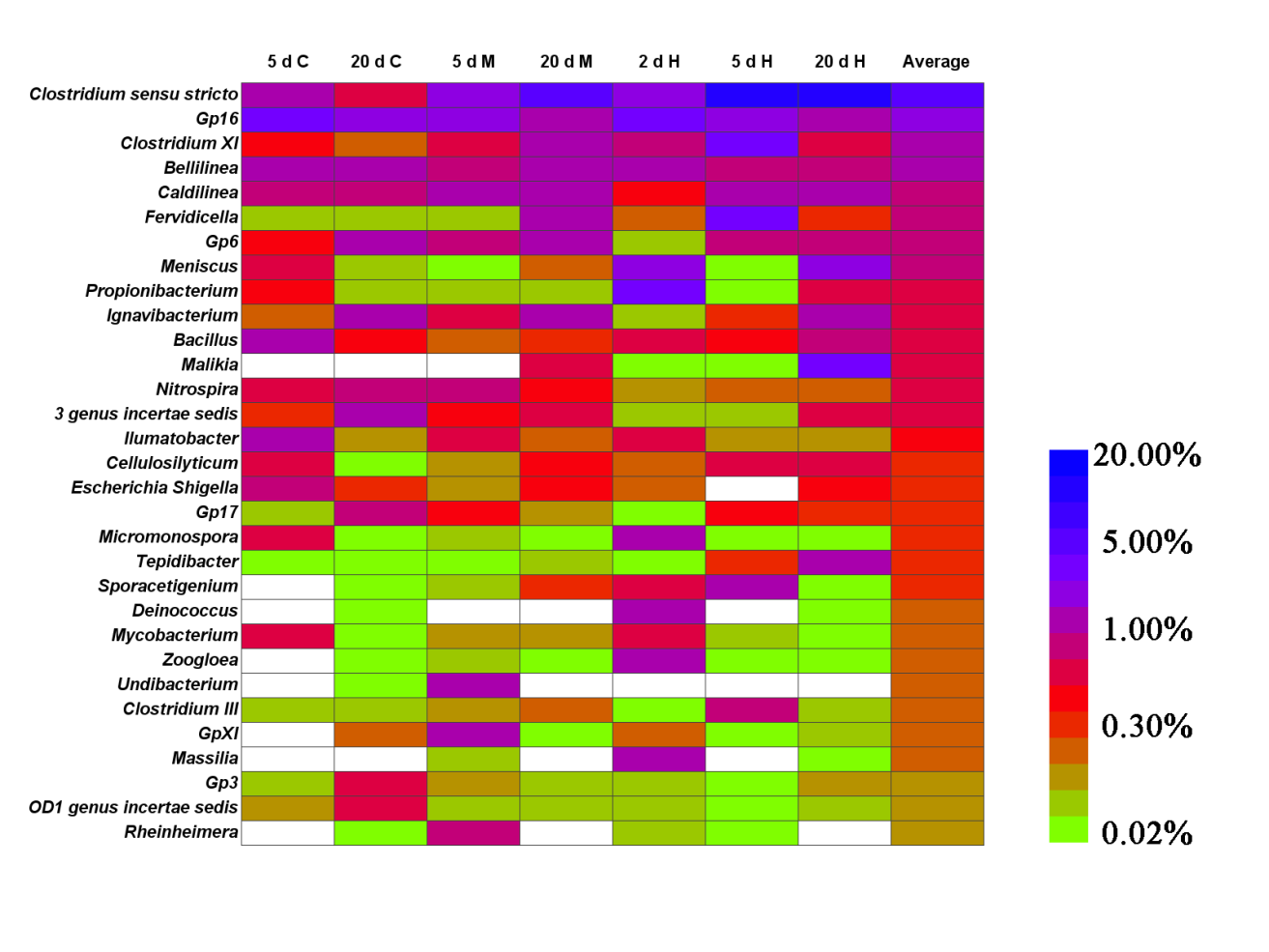


**S2 Fig. Relative abundance of the top 10 genera in each sediments sample of different treatments. C, control treatment without addition of *Microcystis*; M, moderate *Microcystis* biomass treatment; H, High *Microcystis* biomass treatment.**
