# Supplementary material for: Variation of bacterial communities in water and sediments during the decomposition of Microcystis biomass
Source: PLoS One. 2017 Apr 24;12(4):e0176397. doi: 10.1371/journal.pone.0176397 (PMC5402945; doi:10.1371/journal.pone.0176397)
Supplement: S1 Table — (DOCX) [file pone.0176397.s003.docx]

**S1 Table. Relative abundance of all detected bacterial phyla/subphyla in water of different treatments.**

| **Bacterial phyla/subphyla** | **0 d C** | **20 d C** | **5 d M** | **20 d M** | **2 d H** | **5 d H** | **10 d H** | **20 d H** | **40 d H** | **Average** |
| --- | --- | --- | --- | --- | --- | --- | --- | --- | --- | --- |
| *Alphaproteobacteria* | 38.37 | 16.67 | 10.28 | 12.97 | 2.69 | 3.94 | 16.87 | 49.04 | 3.53 | 17.15 |
| *Betaproteobacteria* | 35.69 | 25.11 | 56.44 | 52.04 | 36.08 | 61.60 | 57.44 | 19.12 | 54.38 | 44.21 |
| *Deltaproteobacteria* | 0.29 | 2.94 | 0.80 | 0.81 | 0.00 | 0.20 | 0.42 | 1.44 | 1.38 | 0.92 |
| *Epsilonproteobacteria* | 0.03 | 4.05 | 0.04 | 3.96 | 0.00 | 0.00 | 0.12 | 8.53 | 1.02 | 1.97 |
| *Gammaproteobacteria* | 0.64 | 2.45 | 1.51 | 2.38 | 8.71 | 0.50 | 9.20 | 0.13 | 1.38 | 2.99 |
| *Acidobacteria* | 0.58 | 0.85 | 0.44 | 0.04 | 0.00 | 0.05 | 0.04 | 0.02 | 0.14 | 0.24 |
| *Actinobacteria* | 3.83 | 2.81 | 3.67 | 1.21 | 0.00 | 0.10 | 0.27 | 0.09 | 5.43 | 1.93 |
| *Armatimonadetes* | 0.35 | 0.16 | 0.00 | 0.00 | 0.00 | 0.00 | 0.00 | 0.29 | 0.04 | 0.09 |
| *Bacteroidetes* | 12.56 | 15.10 | 14.23 | 12.28 | 0.24 | 9.48 | 1.96 | 9.84 | 2.15 | 8.65 |
| *Chlorobi* | 0.13 | 0.00 | 0.00 | 0.00 | 0.00 | 0.00 | 0.00 | 0.00 | 0.04 | 0.02 |
| *Chloroflexi* | 0.89 | 0.52 | 0.04 | 0.12 | 0.00 | 0.05 | 0.00 | 0.00 | 0.11 | 0.19 |
| *Cyanobacteria/Chloroplast* | 0.38 | 0.13 | 0.16 | 0.32 | 0.00 | 0.00 | 1.88 | 0.00 | 0.00 | 0.32 |
| *Deinococcus-Thermus* | 0.06 | 0.00 | 0.00 | 0.00 | 0.00 | 0.00 | 0.00 | 0.00 | 0.00 | 0.01 |
| ***Firmicutes*** | **0.54** | **1.18** | **9.41** | **0.73** | **51.61** | **23.29** | **4.52** | **3.60** | **2.08** | **10.77** |
| *Fusobacteria* | 0.03 | 0.00 | 0.08 | 0.00 | 0.20 | 0.05 | 0.00 | 0.00 | 0.00 | 0.04 |
| *Gemmatimonadetes* | 0.06 | 0.03 | 0.00 | 0.00 | 0.00 | 0.00 | 0.00 | 0.02 | 0.00 | 0.01 |
| *Lentisphaerae* | 0.00 | 0.23 | 0.00 | 0.04 | 0.00 | 0.00 | 3.95 | 2.64 | 0.07 | 0.77 |
| *Nitrospira* | 0.35 | 0.00 | 0.00 | 0.00 | 0.00 | 0.00 | 0.00 | 0.00 | 0.18 | 0.06 |
| *Planctomycetes* | 0.16 | 2.68 | 0.04 | 0.40 | 0.00 | 0.00 | 0.00 | 0.00 | 0.07 | 0.37 |
| *Spirochaetes* | 0.00 | 3.14 | 0.00 | 11.88 | 0.00 | 0.00 | 0.27 | 0.76 | 0.32 | 1.82 |
| *Verrucomicrobia* | 0.54 | 1.18 | 0.08 | 0.04 | 0.00 | 0.00 | 0.00 | 0.00 | 0.14 | 0.22 |
| *BRC1* | 0.00 | 0.03 | 0.00 | 0.00 | 0.00 | 0.00 | 0.04 | 0.02 | 0.00 | 0.01 |
| *OD1* | 0.06 | 1.77 | 0.00 | 0.04 | 0.00 | 0.00 | 0.04 | 2.40 | 0.42 | 0.53 |
| *OP11* | 0.03 | 0.00 | 0.00 | 0.00 | 0.00 | 0.00 | 0.00 | 0.00 | 0.00 | 0.00 |
| *TM7* | 0.03 | 0.00 | 0.08 | 0.12 | 0.00 | 0.00 | 0.00 | 0.00 | 0.00 | 0.03 |
| Unclassified bacteria | 4.38 | 18.96 | 2.71 | 0.61 | 0.48 | 0.75 | 2.99 | 2.04 | 27.13 | 6.67 |

C, control treatment without addition of *Microcystis*; M, moderate *Microcystis* biomass treatment; H, High *Microcystis* biomass treatment.
