# Supplementary material for: Variation of bacterial communities in water and sediments during the decomposition of Microcystis biomass
Source: PLoS One. 2017 Apr 24;12(4):e0176397. doi: 10.1371/journal.pone.0176397 (PMC5402945; doi:10.1371/journal.pone.0176397)
Supplement: S2 Table — (DOCX) [file pone.0176397.s004.docx]

**S2 Table. Relative abundance of all detected bacterial phyla/subphyla in sediments of different treatments.**

| **Bacterial phyla/subphyla** | **5 d C** | **20 d C** | **5 d M** | **20 d M** | **2 d H** | **5 d H** | **20 d H** | **Average** |
| --- | --- | --- | --- | --- | --- | --- | --- | --- |
| *Alphaproteobacteria* | 0.63 | 2.18 | 2.65 | 0.79 | 4.04 | 0.68 | 0.74 | 1.67 |
| *Betaproteobacteria* | 4.80 | 7.79 | 11.86 | 10.78 | 5.78 | 4.93 | 13.55 | 8.50 |
| *Deltaproteobacteria* | 1.31 | 8.10 | 2.82 | 4.58 | 1.07 | 4.08 | 3.96 | 3.70 |
| *Epsilonproteobacteria* | 0.00 | 0.48 | 0.08 | 0.00 | 0.03 | 0.00 | 0.03 | 0.09 |
| *Gammaproteobacteria* | 6.39 | 5.59 | 9.04 | 11.02 | 2.99 | 6.12 | 9.60 | 7.25 |
| *Acidobacteria* | 4.98 | 6.87 | 6.82 | 4.18 | 4.13 | 4.42 | 4.09 | 5.07 |
| *Actinobacteria* | 25.56 | 6.28 | 8.46 | 4.07 | 27.53 | 5.22 | 4.11 | 11.60 |
| *Armatimonadetes* | 0.19 | 0.19 | 0.40 | 0.11 | 0.13 | 0.05 | 0.13 | 0.17 |
| *Bacteroidetes* | 1.51 | 2.61 | 8.40 | 3.45 | 4.61 | 2.28 | 5.61 | 4.07 |
| *Chlamydiae* | 0.00 | 0.00 | 0.00 | 0.00 | 0.01 | 0.00 | 0.00 | 0.00 |
| *Chlorobi* | 0.31 | 1.17 | 0.81 | 1.42 | 0.05 | 0.32 | 1.26 | 0.76 |
| *Chloroflexi* | 21.26 | 17.41 | 16.74 | 19.54 | 14.38 | 14.65 | 13.79 | 16.82 |
| *Cyanobacteria/Chloroplast* | 0.62 | 1.13 | 1.28 | 0.30 | 1.42 | 0.43 | 0.30 | 0.78 |
| ***Firmicutes*** | **7.89** | **3.92** | **9.54** | **15.65** | **11.39** | **37.81** | **21.97** | **15.45** |
| *Deferribacteres* | 0.00 | 0.00 | 0.00 | 0.06 | 0.00 | 0.01 | 0.02 | 0.01 |
| *Deinococcus-Thermus* | 0.00 | 0.02 | 0.00 | 0.00 | 1.85 | 0.00 | 0.04 | 0.27 |
| *Elusimicrobia* | 0.00 | 0.00 | 0.00 | 0.01 | 0.00 | 0.00 | 0.00 | 0.00 |
| *Fusobacteria* | 0.00 | 0.01 | 0.04 | 0.00 | 0.06 | 0.04 | 0.00 | 0.02 |
| *Gemmatimonadetes* | 0.08 | 0.52 | 0.17 | 0.11 | 0.06 | 0.26 | 0.04 | 0.18 |
| *Lentisphaerae* | 0.06 | 0.05 | 0.00 | 0.01 | 0.00 | 0.02 | 0.00 | 0.02 |
| *Nitrospira* | 0.67 | 1.00 | 0.95 | 0.41 | 0.16 | 0.24 | 0.24 | 0.52 |
| *Planctomycetes* | 0.20 | 0.66 | 0.13 | 0.08 | 0.05 | 0.11 | 0.11 | 0.19 |
| *Spirochaetes* | 0.00 | 0.16 | 0.06 | 0.38 | 0.00 | 0.05 | 0.17 | 0.12 |
| *Verrucomicrobia* | 0.54 | 1.78 | 0.46 | 1.09 | 0.26 | 0.10 | 0.91 | 0.73 |
| *BRC1* | 0.00 | 0.02 | 0.00 | 0.00 | 0.00 | 0.00 | 0.00 | 0.00 |
| *OD1* | 0.17 | 0.76 | 0.10 | 0.07 | 0.10 | 0.01 | 0.06 | 0.18 |
| *OP11* | 0.00 | 0.05 | 0.05 | 0.06 | 0.00 | 0.00 | 0.02 | 0.03 |
| *SR1* | 0.00 | 0.01 | 0.00 | 0.00 | 0.01 | 0.00 | 0.00 | 0.00 |
| *TM7* | 0.17 | 0.19 | 0.10 | 0.15 | 0.19 | 0.08 | 0.04 | 0.13 |
| *WS3* | 0.06 | 0.50 | 0.13 | 0.06 | 0.01 | 0.03 | 0.12 | 0.13 |
| Unclassified bacteria | 22.60 | 30.55 | 18.93 | 21.62 | 19.68 | 18.05 | 19.10 | 21.50 |

C, control treatment without addition of *Microcystis*; M, moderate *Microcystis* biomass treatment; H, High *Microcystis* biomass treatment.
